# Increased cannabis use in pregnant women during COVID-19 pandemic

**DOI:** 10.15190/d.2022.7

**Published:** 2022-06-30

**Authors:** Arjola Agolli, Olsi Agolli, Selia Chowdhury, Vallabh Shet, Johanna S. Canenguez Benitez, Niharika Bheemisetty, Madeeha Subhan Waleed

**Affiliations:** ^1^Tirana University of Medicine, Albania; ^2^Dhaka Medical College, Bangladesh; ^3^Bangalore Medical College and Research Institute, Karnataka, India; ^4^University of El Salvador, El Salvador; ^5^Kurnool medical college, Andhra Pradesh, India; ^6^Lower Bucks Hospital, Bristol, PA, USA

**Keywords:** Cannabis, COVID-19, pregnancy, marijuana, fetal development.

## Abstract

Almost one in every 20 pregnant women self-reports marijuana use during pregnancy. During the COVID-19 pandemic, this number has risen to 1 in 6 pregnant women. Some of the main factors associated with cannabis use during pregnancy and lactation are management of chronic conditions, sensation-seeking, dealing with stress, and other conditions related to pregnancy. The action of cannabis on endocannabinoid receptors might cause poor blastocyst implantation, inhibition of decidualization, compromised placentation, miscarriage and poor embryo development.The children born to mothers who used cannabis during pregnancy manifested higher aggression, anxiety, hyperactivity, and higher levels of the hormone cortisol, compared to children of non-cannabis users. In this review we summarize the effects of cannabis use on fetal development during the COVID-19 pandemic based on the existing published peer-reviewed scientific literature. The COVID-19 pandemic has served as an additional stimulus that has increased cannabis use among pregnant women. Prenatal cannabis use is associated with health risks for the mother and child. Cannabis use in pregnant mothers is associated with low infant birth weight and potential negative neurodevelopmental effects in the offspring. It remains unclear how long these changes will persist in the affected children. It is essential that clinicians educate pregnant women about the harm of prenatal cannabis use, improve strategies to support women at risk, and create new intervention strategies to help them stop using cannabis.

## SUMMARY

*1. Introduction*

*2. Factors that make women susceptible to substance use during pregnancy*

*3. How does cannabis affect child and maternal health if used during pregnancy?*

*3.1* *Effects of cannabis on fetal development.*

*3.2* *Physiopathology of the endocannabinoid system, and its disruption by Cannabis.*

*3.3* *Hypothesis about correlations between cannabis use during pregnancy and autism*

*4. Conclusion*

## 1. Introduction

Cannabis, also known as marijuana, is the most common illegal drug used in the United States (USA). As of June 2022, a total of 38 states in the USA have legalized cannabis for medical or recreational purposes. Cannabis use during pregnancy is rising, along with the potential for abuse or dependence^1^. According to Centers for Disease Control and Prevention data, almost 48.2 million people living in United States have used marijuana at least once during year 2019. Almost 3 out of 10 people who use marijuana are unable to stop using it. It is reported to affect the brain, especially brain areas responsible for coordination, emotion, memory, learning, attention, decision-making, and reaction time. Infants, children, and teens can be more prone to adverse effects of cannabis due to their developing brain^2^. During history, cannabis has been used for spiritual, medical, and recreational purposes for over 5,000 years^3^. According to Volkow et al., its use among pregnant women has increased in the USA, between 2002 and 2017. It was reported to vary from 3.4% to 7.0% among pregnant women overall during the reported period of time, while it has increased from 5.7% to 12.1% during the first trimester^4^.

**Figure 1:**
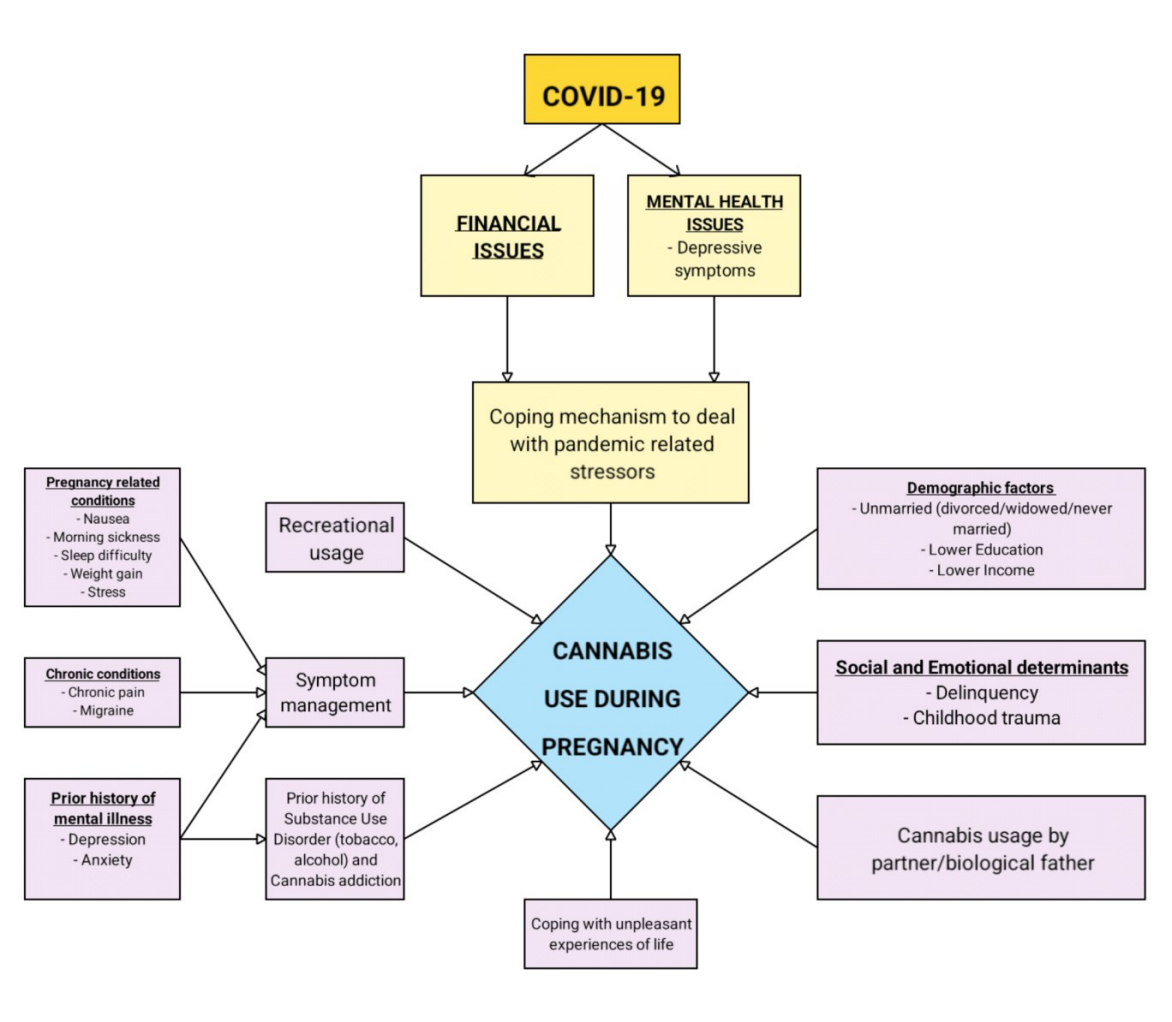
Risk factors for cannabis use during pregnancy

In addition, most studies found that a self-reported prevalence of cannabis use during pregnancy varied from 2% to 5%. Pregnant women report multiple reasons for utilizing cannabis during pregnancy^5^. They mention its use to cope with symptoms related to pregnancy (e.g., weight gain, insomnia, nausea) and to treat existing chronic health conditions. In addition, they use it for improving psychological well-being and for recreational purposes^6^. Pregnant women report using cannabis to relieve anxiety and stress. It has been reported that prenatal cannabis use may have arisen during the COVID-19 pandemic due to pregnant women facing different COVID-related stressors: financial and psychosocial distress, social isolation, etc^7^(Figure 1).

Cannabis usage during pregnancy is on the rise. It is a crucial health concern, since marijuana has the potential to harm both the mother and her offspring^8^. Of note, about 34 to 60 percent of cannabis users continue cannabis even during pregnancy because of a misconception that its usage is safe. The finding of a recent study shows that 18.1% of pregnant women who had used cannabis during the previous year satisfied the criteria for marijuana misuse, dependence, or both^5^. In a nationally representative research study among 18 to 44 years old child-bearing age women, analyzed from 2002 through 2014, it was found that the adjusted prevalence of past-month marijuana use changed from 2.37 percent in 2002 to 3.85 percent in 2014 in the USA^9^. According to the CDC, about one in every 20 pregnant women self-reports marijuana use during pregnancy^2^. According to a recent study, this number has risen to 1 in every 6 pregnant women^3^.

Cannabis use has become more frequent in pregnant patients since the pandemic started. For example, a study conducted by the Research Division in Kaiser Permanente Northern California in 2021 demonstrated an increase from 6.75% to 8.14%. In addition, an increase of 25% in prenatal cannabis use was reported during the pandemic versus pre-pandemic^7^. This increase is worrying, considering the consequences for the offspring of these pregnant patients. Most of the consequences are irreversible, and they can severely affect the newborns, such as neurodevelopmental defects and low birth weight^5^. Furthermore, the pandemic has demonstrated its negative effect on mental health, negatively contributing to increased substance abuse, including cannabis. The social isolation obligated by the circumstances in the COVID-19 pandemic age has significantly increased mood disorders, such as depression and anxiety, leading to increased cannabis use during pregnancy^10^.

## 2. Factors that make women susceptible to substance use during pregnancy

Different research studies have produced heterogeneous results, which is likely due to variances in sample demographics, study designs, and cultural differences in the geographical places where these investigations are conducted^10-14^.

Kar et al. conducted a systematic review on substance and alcohol use in pregnancy during the COVID-19 pandemic. The results of the survey were evaluated as part of a larger research of 7470 pregnant women in Canada. 6.7 percent of participants said they used alcohol during pregnancy, 4.9 percent said they used cigarettes, 4.3 percent said they used cannabis, 0.3 percent said they used illegal drugs and 2.6 percent said they used several substances. The study showed that cannabis and/or cigarette use, as well as co-use of drugs, were linked to higher depressive symptoms and financial issues. There were no links between alcohol use and mental health or COVID-19 issues. More tobacco, cannabis, and drug co-use were linked to depression symptoms and pandemic-related financial issues^10^.

In addition, another recent study reports several categories of reasons why people use cannabis during pregnancy and lactation: symptom management of chronic conditions, sensation-seeking for fun and enjoyment and conditions related to pregnancy. In addition, cannabis it is used for coping with the unpleasant experiences in life^6^.

In the study conducted in Rotterdam, El Marroun et al. found no clear connection between cannabis usage during pregnancy and demographic variables such as age, ethnicity, or the presence of psychopathology. However, there is a clear link between cannabis usage by their biological father or their partner and cannabis use by pregnant women. It seems that a history of delinquency and childhood trauma might also be associated with it. Religion was regarded as a defensive factor against harm. In this sample, 32% of women used cannabis before pregnancy, 29% used cannabis before and during pregnancy, but only 6% of women continued to use cannabis during pregnancy. This last group had a lower degree of education. They also discovered that women who have a history of cannabis addiction are 2.77 times more likely to continue using it during pregnancy. Also, women who use cannabis often (daily or weekly) are more likely to do so versus women who use it monthly. COVID-19-specific financial difficulties predicted more substance use in pregnancy^8^.

The women using cannabis were also likely to use tobacco.Cannabis use was also linked to having a mental illness other than the substance addiction as well as not having completed high school^11^.Another study performed in the USA by Martin et al., identified a link between cannabis usage during pregnancy and other specific traits, such as being young, unmarried, and non-Hispanic white^12^. Some women self-reported the use of cannabis with the intention to relieve severe nausea associated with pregnancy. However, there is no evidence supporting this suggestion, and it is not typically recommended^13,14^.

Knowledge of these above stated factors linked to cannabis usage during pregnancy might be important in identifying future mothers at risk and providing them with accurate information about the risks of prenatal cannabis exposure^7^. To minimize prenatal substance use and prevent poor perinatal and long-term neurodevelopmental outcomes for children, it is critical to retain access to perinatal, mental health, and financial services during the COVID-19 pandemic^10^.

## 3. How can cannabis affect child and maternal health if used during pregnancy?

Cannabis binds to cannabinoid receptors, increases fluidity of cell membranes, causes changes in dopamine in brain reward regions, modulates the γ-amino butyric acid system, causes alteration of neurotransmitters and prostaglandins, and inhibits of calcium uptake by synaptosomes. Two endogenous cannabinoid receptors, CB1 (Cannabinoid Receptor Type 1) mainly found in the brain and CB2 (Cannabinoid Receptor Type 2) only found in peripheral tissues, have been reported. When used during pregnancy, cannabis may reduce the size of the fetus and the birth weight. In children whose mothers used marijuana before or during gestation, it also causes a 10-fold increase in the risk of non-lymphoblastic leukemia and it can increase the risk of chromosomal damage (including breakage and translocation), damaging mainly the somatic cells.

The endocannabinoid system, is a complex system, involving “the main endogenous ligands anandamide and 2-arachidonoyl glycerol, the cannabinoid receptors CB1 and CB2 and the biosynthetic and degrading enzymes. Evidence shows that the endocannabinoid system plays an essential role in reproduction, from egg fertilization to parturition”^15^. As a result, the “alterations in this system, either by recreation/therapeutic use of cannabis or deregulation of the endogenous cannabinoids, might lead to retardation in embryo development, poor blastocyst implantation, inhibition of decidualization”, miscarriage and compromised placentation^15^. Prenatal cannabis exposure can affect *in utero* development of the nervous system by impacting the formation and functions of neuronal circuitries through action on cannabinoid receptors. By prolonging the ‘switched-on’ period of cannabinoid receptors, it can hijack endocannabinoid signals to evoke molecular rearrangements. This can lead to the erroneous wiring of neuronal networks^16^.

A total of 100 005 pregnancies (95 412 women), with a mean age of 31 years have been recently studied in Northern California. During the pandemic, patients completed toxicology testing slightly earlier in their pregnancies (before pandemic mean, 8.51 weeks’ gestation; during pandemic mean, 8.04 weeks’ gestation). Before the pandemic, the standardized rate of prenatal cannabis use was 6.75% of pregnancies (95% CI, 6.55%-6.95%). However, that rate increased to 8.14% of pregnancies (95% CI, 7.85%-8.43%) during the pandemic. They found that prenatal cannabis use increased by 25% (95% CI, 12%-40% during the pandemic over prenatal cannabis use during the 15 months before the pandemic^7^.

### 3.1 Effects of cannabis on fetal development

The effects of maternal cannabis use on “psychosocial and physiological measures in young children along with the potential relevance of the in-utero environment reflected in the placental transcriptome. Children (∼3 to 6 years old) were assessed for hair hormone levels, neurobehavioral traits on the Behavioral Assessment System for Children (BASC-2) survey, and heart rate variability (HRV) at rest and during auditory startle”^17^. For a subset of the placental specimens collected at birth of children with behavioral assessments, RNA sequencing was performed. Analysis of the hair revealed higher concentration of the hormone cortisol in children of mothers who used cannabis during pregnancy. In addition, cannabis use in pregnant women was associated with greater aggression, anxiety and hyperactivity. The risk for problems related with anxiety in early childhood may be associated/related with a relationship between cannabis use in pregnant women and the immune system gene network response in the placenta^17^.

The incidence of intellectual disability and learning disorders was reported to be higher among offspring of mothers who use cannabis during pregnancy^18^.

Stress may be modulated by the cannabinoid signaling, this being a reason for people to use cannabis in order to decrease anxiety and relax. A higher rate of aggression, anxiety, hyperactivity and higher levels of the hormone cortisol, was observed among the children whose mothers consumed cannabis versus children of non-cannabis users. Maternal cannabis use was also associated with a reduction in the high-frequency component of heart rate variability which normally reflects increased stress sensitivity. In addition, maternal cannabis use is associated with lower expression of immune activating genes in the placenta, such as certain pro-inflammatory cytokines, important genes in anti-pathogen protection. Cannabis can inhibit a few placental immune-gene networks, which is related to the prediction for a higher anxiety in the children^19^. Experimental studies performed in rats, also show that maternal exposure to synthetic cannabinoid affects the development of the immune system. This prenatal exposure caused reduction in the T-helper subpopulation in the spleen and decrease in the ratio of T helper/ cytotoxic T cells in the peripheral blood of adult offspring^20^. According to another study, during pregnancy 4.3 % reported the use of cannabis and 0.3 % the use of illicit drugs. Higher cannabis, and/or tobacco use and co-use of other substances is associated with depression symptoms and financial difficulties^10^.

### 3.2 Physiopathology of the endocannabinoid system, and its disruption by Cannabis

Following maternal cannabis use, the psychoactive metabolite delta-9-tetrahydrocannabinol (THC) crosses the placenta and enters the fetus. Due to the lipophilic nature of cannabinoids, they bypass the blood brain barrier and act on the developing fetal brain^3^. THC acts on cannabinoid receptors (CB1) in the brain, which is part of the endogenous cannabinoid system 1. This system plays a critical role in nervous system functioning and development during pregnancy, contributing to cell differentiation, neuronal proliferation and migration, and synaptogenesis. These cannabinoid 1 receptors are mainly expressed in mesocorticolimbic brain structures during prenatal development. In utero cannabis exposure can lead to permanent developmental disruption of the mesocorticolimbic system which is regulated by dopamine, predisposing the child to various psychiatric and substance use disorders. THC also inhibits the production of γ-Aminobutyric acid (GABA), affecting inhibitory afferent neurons that relay onto dopaminergic neuron dendrites^21^. These de-inhibited dopaminergic neurons then show increased activity, which result in the perceived effects of cannabis in neonates such as a neonatal abstinence like syndrome with tremors, irritability and a heightened startle reflex^22^.

This synaptic modulation is disrupted by cannabis use, having functional implications in reward processing, motor function, memory, cognition, analgesia and development of addictive behaviour^23^.

**Figure 2:**
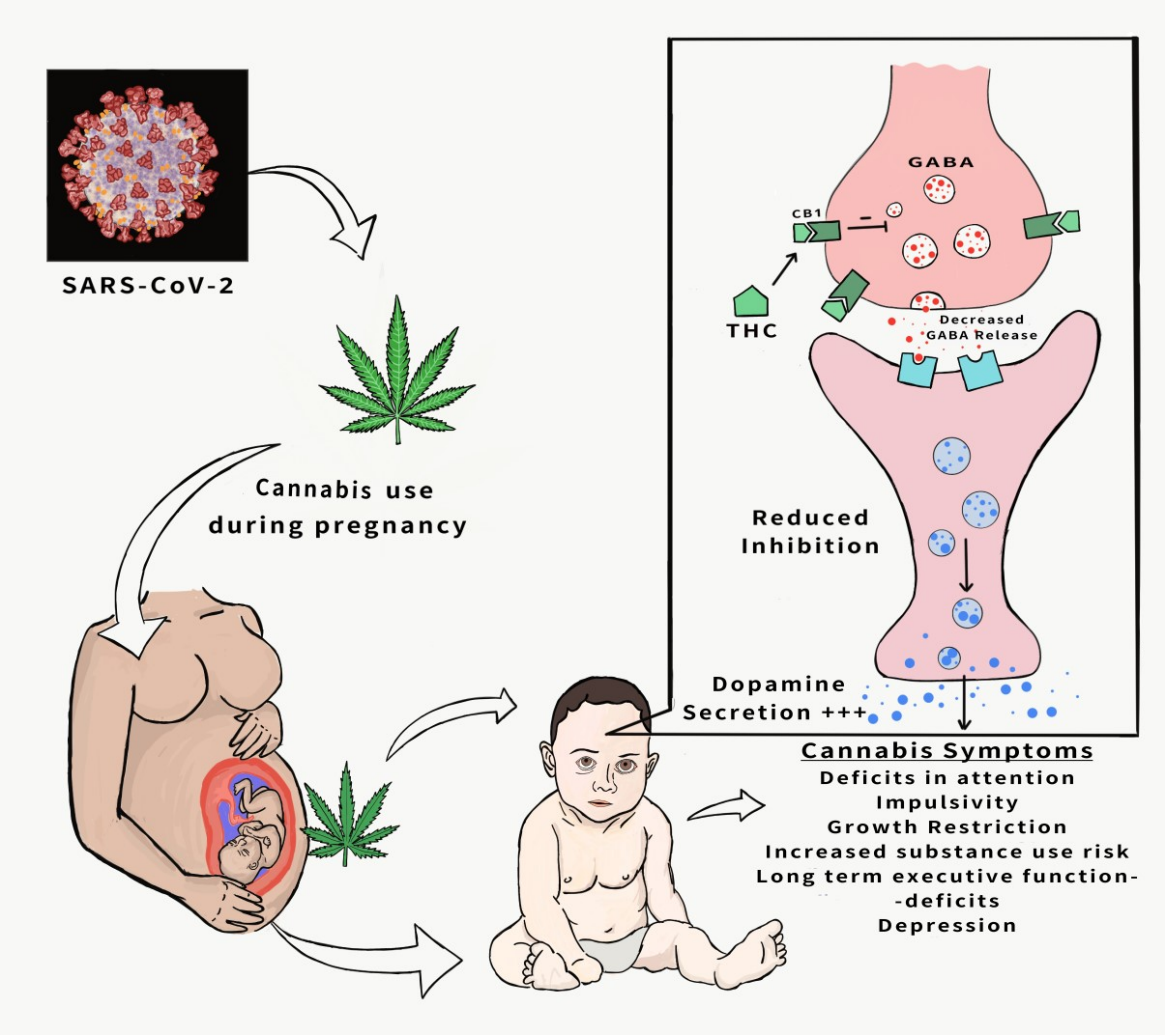
Physiopathology of the endocannabinoid system, and its disruption by cannabis

Some studies that have assessed the effects of maternal cannabis during pregnancy have shown an association with early pregnancy loss, stillbirth, poor fetal growth and other adverse newborn outcomes. In other studies, mixed results have been reported. Due to the presence of confounding factors related to polysubstance use, reliability of self-reporting and availability of drug testing these studies are limited^24^(Figure 2).

### 3.3 Hypothesis about correlations between cannabis use during pregnancy and autism

Autism spectrum disorder (ASD) is a complicated neurological disorder with impaired sensory inputs, abnormal social interactions, and certain behavior repetition^25^. Various genetic and environmental factors are responsible for causing this condition^26^. One of the factors contributing to the development of ASD is the use of cannabis during pregnancy. This leads to various neurochemical changes in the brain leading to ASD. The active component of cannabis is technically THC. This constituent can cross the placenta, transverse the placental blood-brain barrier, and can disrupt the innate cannabinoid signaling. THC adheres to the CB1 receptors and upregulates the production of dopamine by causing the suppression of the GABAergic neurons in the brain that lead to suppression of the CB1 receptors reciprocally. Thus, impairing the normal neurological development of the fetal brain and leading to developmental deficits and neurocognitive disorders in the developing fetal brain^27,28^.

The incidence of a child exposed to cannabis has been reported to be 4/1000 person-years as compared to 2.42/1000 in unexposed children^18^. Peri-pregnancy cannabis use was reported for 5.2% of ASD, 3.2% of developmental delays/disorders (DD), and 4.4% of population controls (POP) children. Another study indicated that 5.2 percent of the children with ASD and 3.2 percent of developmental delays had maternal exposure to cannabis^29^.

Also, in a recent study by Corsi, D.J et al, found an association between maternal cannabis use in pregnancy and the incidence of autism spectrum disorder in the offspring. The incidence of autism spectrum disorder diagnosis was reported as 4.00 per 1,000 person-years among children with exposure versus 2.42 among unexposed children. In addition, the fully adjusted hazard ratio was 1.51 (95% confidence interval: 1.17–1.96) in the matched cohort^18^.

Currently, there is no cure for ASD and the condition causes a profound financial and emotional effect on patients' families. A proper management team is required involving the primary care physician, obstetrician, pediatrician, and drug dependence specialists for the management of the pregnant patient with cannabis dependence. Autism is a devastating condition, and it is critical to educate women regarding the consequences of cannabis abuse during pregnancy^30^.

The long-term effects of cannabis use during pregnancy on newborn is still unclear. A total of 24,874 women were included and analyzed during a 7-years period study in Australia. The study participants provided information about cannabis use, and their birth outcomes data were available. They reported an association between smoking cannabis during pregnancy and a lower birth weight, shorter birth length and a higher rate of premature birth^31^. In another recent study done in Nova Scotia from 2004 to 2021, was reported that cannabis use in pregnancy increased from 1.3% to 7.5% over the study period with no appreciable change in slope after legalization in 2018. The authors also reported cannabis use during pregnancy to be associated with early postnatal complications and reduced fetal growth^32^. However, a cohort study by Fine JD et al, which used data from the Adolescent Brain Cognitive Development (ABCD) study among 4361 children born between 2005 and 2008 showed that prenatal cannabis exposure after maternal knowledge of pregnancy may be associated with an increase in psychosis proneness in middle childhood^33^. A longitudinal study by Sonon KE et al at the University of Pittsburgh, involved 589 mother-child pairs followed up from the fourth gestational month up to when the offspring were 22 years of age. This study showed that prenatal marijuana exposure predicted marijuana use in the offspring at 14 years and 22 years, after controlling for covariates^34^.

## 4. Conclusions

Covid-19 pandemic has served as an additional stimulus that has increased cannabis use among pregnant women. Prenatal cannabis use is associated with health risks, in mother and child. Children born by cannabis using mothers are associated with low infant birth weight and potential effects on offspring neurodevelopment. It is essential that clinicians educate pregnant women about the harms of prenatal cannabis use, elaborate support for women at risk, implement intervention strategies to help them stop using cannabis and offer psychosocial support.

## KEY POINTS

**◊** *About one in every 20 pregnant women self-reports cannabis use during pregnancy*

**◊** *Cannabis use among pregnant women has risen during COVID-19 pandemic*

**◊** *Maternal cannabis use during pregnancy has been reported to be associated with poor perinatal and long-term neurodevelopmental outcomes for children*

**◊** *It remains unclear how long the cannabis use related changes will last in affected children*
